# Ameliorative Effect of Neem Leaf and Pomegranate Peel Extracts in Coccidial Infections in New Zealand and V-Line Rabbits: Performance, Intestinal Health, Oocyst Shedding, Carcass Traits, and Effect on Economic Measures

**DOI:** 10.3390/ani11082441

**Published:** 2021-08-19

**Authors:** Liza S. Mohammed, Eman A. Sallam, Sawsan S. El basuni, Amany S. Eldiarby, Mohamed Mohamed Soliman, Salama Mostafa Aboelenin, Seham F. Shehata

**Affiliations:** 1 Veterinary Economics and Farm Management, Department of Animal Wealth Development, Faculty of Veterinary Medicine, Benha University, Benha 13736, Egypt; seham.shehata@fvtm.bu.edu.eg; 2 Animal and Poultry Production, Department of Animal Wealth Development, Faculty of Veterinary Medicine, Benha University, Benha 13736, Egypt; eman.salam@fvtm.bu.edu.eg; 3 Avian and Rabbit Diseases Department, Faculty of Veterinary Medicine, Benha University, Benha 13736, Egypt; sowsan.mohamed@fvtm.bu.edu.eg; 4 Parasitology Department, Veterinary Teaching Hospital, Faculty of Veterinary Medicine, Benha University, Benha 13736, Egypt; amany.salah@fvtm.bu.edu.eg; 5 Clinical Laboratory Sciences Department, Turabah University College, Taif University, P.O. Box 11099, Taif 21944, Saudi Arabia; mmsoliman@tu.edu.sa; 6 Biology Department, Turabah University College, Taif University, P.O. Box 11099, Taif 21944, Saudi Arabia; s.aboelenin@tu.edu.sa

**Keywords:** coccidial infection, neem, pomegranate, New Zealand rabbit, V-line rabbit

## Abstract

**Simple Summary:**

Coccidiosis, one of the most contagious diseases among domestic rabbits, negatively affects production and results in massive economic losses. This study evaluates the therapeutic efficacy of treatment with aqueous neem leaf extract and ethanolic pomegranate peel extract (PPE) individually and in combination on the intestinal coccidiosis caused by *Eimeria* spp. in New Zealand white and V-line (VL) rabbits. Rabbits from two breeds were divided into ten equal groups (five groups each for NZ and VL). All rabbits were inoculated with *Eimeria* spp. oocysts except for the rabbits in the first group (G1) (negative control). The remaining groups were: G2, positive control, G3, treated with neem leaf extract, G4, treated with pomegranate peel extract (PPE), and G5, treated with a combination of neem leaf extract and PPE. Our results showed that the use of neem leaf and/or pomegranate peel extract for both breeds resulted in improved growth performance, a significant reduction in mean oocyst count, no mortalities, an anticoccidial index > 120, and significantly improved economic efficiency measures when compared to the positive control group.

**Abstract:**

Healthy, weaned, coccidial-free male rabbits from two breeds (New Zealand white (NZ) and V-line (VL)) were divided into 10 equal groups (5 groups each for NZ and VL) (3 replicates/group, 6 rabbits/replicate, 18 rabbits/group). All rabbits were inoculated with 5 × 10^4^ *Eimeria* spp. oocysts *(E. intestinalis* (67%), *E. magna* (22%), and *E. media* (11%)) except for the rabbits in the first group (G1), which were inoculated with a sterile solution and served as a negative control. The remaining four groups were treated as follows: G2, no treatment/positive control, G3, treated with neem leaf extract, G4, treated with pomegranate peel extract (PPE), and G5, treated with a combination of neem leaf extract and PPE. For both breeds, our results showed that the use of neem leaf and/or pomegranate peel extract resulted in improved growth performance, with a significant improvement in relative feed conversion ratio (FCR) compared to the positive control groups, which recorded the worst values, as well as a significant (*p* ≤ 0.05) reduction in mean oocyst count compared to the positive control groups. We also observed downregulation of mRNA levels of IL-1βα, IL6, and TNF-α in the herbal treatment groups compared with the mRNA levels of these genes in the positive control groups. Herbal treatment with neem leaf and/or pomegranate peel extracts had positive effects on the NZ and VL rabbits experimentally infected with mixed *Eimeria* species, as evidenced by their healthy appearance, good appetite, no mortalities, an anticoccidial index > 120, and a significantly higher total return and net profit when compared to the positive control groups of both breeds. In NZ rabbits, the treatment with neem leaf extract alone (G3) or in combination with PPE (G5) recorded the most efficient economic anticoccidial activity.

## 1. Introduction

Raising rabbits for their meat has gone a long way towards solving the problem of the global meat shortage and is ranked as the third-largest meat production industry, behind those of beef and poultry, in terms of volume and importance. Moreover, rabbit meat is of excellent quality, rich in protein, and low in fat and calories [1]. According to the Food and Agriculture Organization, in 2017, Egypt ranked as the fourth largest producer of rabbit meat in the world, following China, the Democratic People’s Republic of Korea, and Spain. Its production was estimated at 62,262 tons (3.8% of the global production) that year [2].

Coccidiosis, one of the most contagious diseases among domestic rabbits, negatively affects production and results in massive economic losses [3,4]. The prevalence of coccidial infection among domestic rabbits in Egypt can reach 70% and occurs mainly in mixed infections caused by three different species of intestinal coccidiosis [5].

Intestinal coccidiosis in rabbits is caused by thirteen species in the genus *Eimeria* (*E. irresidua*, *E. magna*, *E. media*, *E. perforans*, *E. intestinalis*, *E. piriformis*, etc.). These parasites can multiply rapidly in the different regions of the intestine, causing mild-to-severe gross and histopathologic lesions depending on the virulence, the infective dose, and the species of *Eimeria*, as well as the age, breed, and immune status of the rabbit [6]. Characteristic symptoms of intestinal coccidiosis include diarrhea, dehydration, listlessness, decreased body weight, poor feed conversion, and mortality [7]. Coccidiosis, an enzootic disease found in many areas of the world including Egypt, is difficult to treat, let alone eradicate. The infective *Eimeria* oocysts are highly resistant to environmental stress and, as a result of inadequate biosecurity measures and improper management practices at the farm level, frequently contaminate feed and water [8,9]. Additionally, the strategies used to control coccidiosis in rabbits have been hampered by the emergence of widespread resistant *Eimeria* strains, no available vaccines for rabbit coccidia, and the toxicity of synthetic drugs/disinfectants for rabbits and humans [6].

The above-described challenges demonstrate the urgent need to find natural alternatives to drugs and disinfectants with the ability to inhibit the growth of *Eimeria*, improve the immune system of rabbits, and increase animal productivity [6,10]. Recently, several plants have been tested for their ability to control coccidiosis [11]. Among these plants, *Azadirachta indica* and *Punica granatum* possess anticoccidial properties and have been used individually in feed to combat avian coccidiosis [12,13]. *Azadirachta indica* (commonly known as neem), which belongs to the family Meliaceae, is one of the most versatile of medicinal plants, with a broad spectrum of biological and pharmacological activities [14]. Neem leaves contain a variety of chemical constituents, including nimbin, nimbanene, 6-desacetylnimbinene, quercetin, nimbandiol, nimbolide, ascorbic acid, n-hexacosanol and amino acid, 7-desacetyl-7-benzoylazadiradione, 7-desacetyl-7-benzoylgedunin, 17-hydroxyazadiradione, and nimbiol [15,16,17], with antibacterial, antifungal, antiviral, anticoccidial, and antimalarial activities [18,19]. Flavonoids in neem leaves, such as nimbin, are considered powerful antioxidants that reduce damage by minimizing the production of reactive oxygen species [20]. In addition, they are strong anti-inflammatory agents with the ability to inhibit prostaglandin biosynthesis, as well as endoperoxides and enzymes such as protein kinases and phosphodiesterase, all of which are involved in inflammation [20,21].

*Punica granatum* (commonly known as pomegranate) has been described as nature’s power fruit and has long been used in folk medicine to treat various diseases due to its potent antioxidant and anti-inflammatory properties [22]. A crude extract of pomegranate fruit peel was effectively used to control *Eimeria tenella* in broiler chickens [13].

Various active ingredients in pomegranate peels, such as tannins, flavonoids, alkaloids, and organic acids, display a host of biological and pharmacological activities. Tannins, such as punicalin, punicalagin, pedunculagin, gallic acid, and casuarinin, are powerful antioxidants [23,24]. Gallagyldilacton, gallic acid, and granatin B are potent anti-inflammatory agents [25]. In the same context, neem leaf extract contains several active components, including nimbin, nimbinene, 6-desacetylnimbiene, nimbandiol, nimbolide, and quercetin [26], all of which are essential for the effects produced by neem. These polyphenols have demonstrated a clear inhibitory effect on inflammatory mediators and pro-inflammatory cytokines via regulation of mytogen activated protein kinases (MAPK) signaling pathways, as well as other effects that are essential for health [27]. In addition, the flavonoids in pomegranate peels have antibacterial, antiviral, antioxidant, anti-inflammatory, and anti-cancer activities [28,29].

Herbal treatments have been shown to provide an effective and economical alternative to prophylactic anticoccidial medications [11]. However, their therapeutic use for coccidiosis control in rabbit production is not yet widespread. Therefore, we carried out this study to evaluate the anticoccidial effects of aqueous neem leaf extract and ethanolic pomegranate peel extract (PPE) individually and in combination. We examined their anti-inflammatory and antioxidant effects in rabbits with respect to intestinal health, cytokine gene expression, oocyte shedding, and fecal scoring. Additionally, we evaluated the deteriorative effect of coccidiosis on rabbit performance and whether these two extracts reduced the deleterious effects and losses of coccidiosis caused by *Eimeria* spp.

## 2. Materials and Methods

This work was conducted according to the ethical standards of the Faculty of Veterinary Medicine, Benha University, Egypt, and approved by the Institutional Animal Care and Use Committee of Benha University and following the guidelines of the National Institute of Health (NIH) in Egypt under ethical number BUFVTM 04-03-21.

### 2.1. Experimental Rabbits

A total of 90 weaned male New Zealand white breed and 90 weaned male V-line breed (VL) rabbits were purchased from a governmental farm in Sharqia, Egypt. The rabbits were in good physical condition and were kept under observation for 7 days with a daily examination of their feces using the concentration flotation technique [30] to confirm that they were coccidial-free. Rabbits accessed water ad libitum and were fed a basal pelleted diet formulated to meet the recommended nutritional requirements for growing rabbits [31]. The diet contains the following: berseem hay 16% crude protein (30%), yellow corn (20%), wheat bran (19.30%), soybean meal 46 (18.80%), Shmer Straw (5.00%), barley grain (4.00%), limestone (1.25%), dicalcium phosphate (0.90%), sodium chloride (0.40%), vitamins and mineral premix (0.30%), and sodium bicarbonate (0.05%). The chemical composition was crude protein (18.11%), digested energy (2600.42 kcal/kg), crude fat (2.06%), crude fiber (13%), lysine (0.92%), methionine + cystine (0.52%), calcium (1.19), total phosphorus (0.60%), and sodium (0.18%). The diet was free from anticoccidial drugs. All rabbits were vaccinated against rabbit hemorrhagic virus. Basic hygienic measures were maintained throughout the experiment.

### 2.2. Collection and Preparation of Eimeria Oocysts

Oocysts of *Eimeria* spp. were isolated from fecal samples from naturally infected rabbits. The collected *Eimeria* oocysts were mixed with 2.5% potassium dichromate solution, incubated at 25 °C, aerated, and examined daily for sporulation [32]. After complete sporulation, the oocysts were centrifugally washed several times until the supernatant became clear; then, the supernatant was removed and stored at 4 °C until further use.

Three rabbits (1 month old) were inoculated orally with 2 mL of a solution containing 5 × 10^4^ sporulated oocysts for *Eimeria* propagation [33]. The inoculated rabbits were examined daily for oocyst shedding, at which time the oocysts were collected for sporulation as described before. The sporulated oocysts were stored at 4 °C until they were used for the challenge in the experiment. The morphometric characters of sporulated oocysts were identified according to the methods of Levine [34] and subsequently counted using the McMaster technique. The stock solution of sporulated oocysts comprised three *Eimeria* species: *E. intestinalis* (67%), *E. magna* (22%), and *E. media* (11%) Figure S1.

### 2.3. Preparation of Herbal Extracts

#### 2.3.1. Aqueous Neem Leaf Extract

Fresh neem leaves (*Azadirachta indica*) were collected from the neem tree at the Faculty of Agriculture, Benha University. The collected leaves were dried at room temperature to prepare them for use in the concentrated aqueous neem leaf extract (4%). In brief, a 40 g aliquot of leaf powder was placed in a non-metallic container and soaked with 1 L of hot boiled distilled water for 5–8 h. The prepared extract was used at a dose of 50 mL/L drinking water according to the methods of Durrani et al. [35].

#### 2.3.2. Ethanolic Pomegranate Peel Extract

Ripe pomegranate (*Punica granatum*) fruit was obtained from the local market, the fruit was peeled, and the peels were collected and dried in the shade. The dried peels were ground into a powder, sieved, and packed in airtight bottles. One kilogram of dried powdered pomegranate peel was extracted successively with 3 L of 70% ethanol and kept at room temperature for 72 h with shaking. The extracts were filtered two times using Whatman No.1 filter paper. The extract was concentrated under reduced pressure at 50 °C and then dried using a rotary evaporator. The dried crude extract was kept at −20 °C in a dark sterile container until further use [36,37].

### 2.4. Experimental Design

The rabbits of each breed (NZ and VL) were labeled using ear and cage tags, weighed individually, and divided into 10 equal groups (5 groups for NZ, 5 groups for VL) (3 replicates/group, 6 rabbits/replicate, 18 rabbits/group). The rabbits in all the groups were inoculated with 5 × 10^4^ *Eimeria* spp. oocysts, except for the first group, G1 (N&V), which was inoculated with a sterile saline solution and served as a negative control. The remaining four groups were treated as follows: Rabbits in the G2 (N&V) groups were not treated and served as a positive control, rabbits in the G3 (N&V) groups were treated with 4% neem leaf extract at dose of 50 mL/L of fresh drinking water for 6 days post-infection (DPI), rabbits in the G4 (N&V) groups were treated with PPE at a dose of 300 mg/kg body weight (BW) for 6 DPI administered by oral gavage, and rabbits in the G5 (N&V) groups were treated with a combination of 4% neem leaf extract and PPE using the same doses as those used in G3 and G4 respectively, for 6 DPI (Table S1). The experimental period of 7 weeks included 3 different stages: the pre-infection period (in the 2nd week of the experiment), the infection and treatment period (in the 3rd week of the experiment), and the post-infection period (2 weeks after treatment in 5th week of the experimental period and 4 weeks after treatment in the 7th week of the experimental period).

### 2.5. Parameters Evaluated to Assess the Efficacy and Anticoccidial Activity of the Herbal Extracts against Intestinal Coccidiosis in Two Different Rabbit Breeds

#### 2.5.1. Growth Performance Parameters

The growth performance parameters for the rabbits were evaluated weekly throughout the experimental period. These included the live body weight (BW) of the rabbits, determined by weighing them individually, and feed intake [38], which was calculated by subtracting the residual feed from the feed offered. Average body weight gain (BWG) for each rabbit was calculated using BWG = BW2 − BW1. Average daily gain was calculated using the formula ADG = BWG ÷ experimental period (days). Mortality was checked daily to allow for adjustments to the feed conversion ratio (FCR). The weekly feed conversion ratio was calculated using FCR = ((F1(g/rabbit/week)) ÷ ((BWG(g/rabbit/week)), as described by Abo-Eid et al. [39], where BW1 = BW at the beginning of the experimental period and BW2 = BW at the end of the experimental period. The relative performance parameters (FCR and BWG) were calculated with regard to the positive and negative control groups as follows:

Relative FCR = (FCR of tested group/FCR of control group) × 100(1)

Relative BWG = (BWG of tested group/BWG of control group) × 100(2)

#### 2.5.2. Parasitological Analysis

In the different groups, freshly voided fecal samples were collected separately at the same time/day and examined daily post-coccidial infection to determine the following items:

##### Fecal Scoring

The fecal consistency was scored on a scale from 0 to 7. A score of 0 indicated normal condition (very hard and dry pellets without residue left on the ground when picked up), through to 7, which indicated severe diarrhea (watery flat feces with no texture, occurring as puddles) [40].

##### Oocyst Shedding

The rabbit groups were kept under control with their feces monitored daily to allow detection of the first day of oocyst shedding [41]. The freshly voided fecal samples from the different groups were collected separately at the same time/day for oocyst counting using the McMaster counting technique [42] and calculated as oocysts per gram of feces (OPG) [43] using the following formula:

OPG = oocyst count × dilution factor × (sample volume/counting chamber volume)(3)

#### 2.5.3. Clinical and Pathological Parameters

##### Clinical Investigation

Rabbits in different groups were observed daily for 14 DPI. The clinical signs (such as loss of appetite, diarrhea, depression, and ruffled hair) and mortalities were recorded. In addition, the relative level of protection (RLP) among the challenged rabbits was determined [44] as follows:

RLP% = 100 − (mortality% of treated groups ÷ mortality% of positive control) × 100(4)

##### Macroscopic Examination and Lesion Scoring

On the 14th DPI, 3 rabbits from each group were humanely slaughtered and the intestines were examined macroscopically. The macroscopic lesion scores for the small and large intestines were assessed using the criteria of Elbahy et al. [45], where a score of 0 signified that there were no evident lesions, while a score of 3 was assigned to severely infected rabbits.

##### Histopathological Examination and Microscopic Lesion Scoring

Jejunal samples from the slaughtered animals were preserved immediately in buffered formalin (10%) and then embedded in paraffin. Sections of paraffin-embedded tissue were stained with hematoxylin-eosin [46] and examined under a light microscope. The histopathological lesions of the jejunum in the different groups were scored on a scale from 0 to 4 according to the nature and extent of the infection [10] and used to calculate the total histological injury score (HIS) according to the method of Dommels [47], using the sum of the inflammatory lesions score (2×), the tissue destruction score, and the tissue reparation score.

##### Anticoccidial Index

To assess the efficacy of the different herbal treatments on the 14th DPI, the anticoccidial index [48] was calculated using the following formula [49]:

ACI = ((Survival% + relative weight gain) − (intestinal lesion index + oocyst index))(5)

where relative weight gain is the percentage of the relative weight gain in the 4th week of the experimental period (on the 14th DPI), the intestinal lesion index is the small intestine lesion score × 10, and the oocyst index is calculated using the formula: ((OPG of treated group ÷ OPG of control positive group) × 100). For the treated groups, an ACI value of below 120 represents no anticoccidial efficacy, a value between 120 and 160 represents low efficacy, a value between 160 and 180 represents medium efficacy, and a value above 180 represents a highly efficient anticoccidial treatment.

#### 2.5.4. Immunological Parameters

To determine the effect of coccidial infection on jejunal cytokine production, including IL-1β, IL6, and TNF-α, and the efficacy of the different herbal treatments on their synthesis, jejunal tissues from the three slaughtered rabbits in the different groups were collected aseptically on the 14th DPI, cut longitudinally, washed with saline, blotted on filter paper, and preserved at −80 °C until RNA extraction. The expression of the IL-1β, IL6, and TNF-α genes was analyzed using real-time PCR with sense and antisense primers. Total cellular RNA was extracted from the tissues using TRIzol (Invitrogen, ThermoFisher Scientific, Carlsbad, CA, USA) (1 mL of TRIzol reagent per 100 mg of tissue sample). The concentration and purity of RNA were determined by measuring the absorbance in a SPECTROstar Nano absorbance plate reader (BMG Lab Tec GmbH, Ortenberg, Germany) at optical density of 260 and 280 nm. RNA integrity was analyzed using a 1% agarose gel stained with 10% ethidium bromide and visualized using a gel documentation system (Gel DOC XR+ System, BIORAD, Hercules, CA, USA). RNA samples were reverse transcribed to cDNA using a High-Capacity cDNA Reverse Transcription Kit (Applied Biosystems, Foster City, CA, USA) according to the manufacturer’s instructions. Five micrograms of total RNA from each sample were reverse transcribed into cDNA. The cDNA preparations were stored frozen at −20 °C until further use. Real-time PCR was performed using an SYBR Green qPCR Master Mix (TOPreal qPCR 2X PreMIX) following the manufacturer’s protocol. PCR was performed in a 20 μL reaction volume containing 10 μL of SYBR Green qPCR Master Mix, 1 μM each of the sense and antisense primers, 1 μL of 1 μg/μL cDNA, and nuclease-free water to a final volume of 20 μL.

Primer sets were as follows: TNF-α, sense (5′-CTGCACTTCAGGGTGATCG-3′) and antisense (5-CTACGTGGGCTAGAGGCTTG-3′); IL-1β, sense (5′-TTGAAGAAGAACCCGTCCTCTG-3′) and antisense (5′-CTCATACGTGCCAGACAACACC-3′); IL6, sense (5′-CTACCGCTTTCCCCACTTCAG-3′) and antisense (5′-TCCTCAGCTCCTTGATGGTCTC-3), and GAPDH as a housekeeping gene, sense (5′-GCCGCTTCTTCTCGTGCAG-3′) and antisense (5′-ATGGATCATTGATGGCGACAACAT-3′).

The real-time PCR cycling program consisted of reverse transcription at 48 °C for 30 min and initial PCR activation at 95 °C for 10 min, followed by 40 cycles of 95 °C for 15 s and 60 °C for 1 min, and a dissociation curve was added to the protocol whenever necessary. A real-time PCR assay was performed using a 7300 real-time PCR system (Applied Biosystems, Foster City, CA, USA). Change in the expression of the genes was calculated from the cycle threshold values (Ct) provided by the real-time PCR using the 2^−ΔΔCt^ calculation, where ΔCt indicates the Ct changes in target genes in comparison to a reference gene (GAPDH; housekeeping gene) [50,51].

#### 2.5.5. Carcass Traits

At the end of the 7th week, 5 rabbits in each group were weighed and then humanely slaughtered. The slaughtered rabbits were bled, and then the skin, viscera, and the distal portion of the legs were removed. Hot carcasses (with the head) were weighed, and visceral organs (intestines, stomach, liver, spleen, and kidney) as well as dressing percentage and relative organ weight were calculated as a percentage of the live body weight [48,52].

#### 2.5.6. Economic Efficiency Parameters

To determine the economic efficiency among the different groups in the experiment, different costs and return parameters were measured as follows.

Cost parameters including total variable cost and total fixed cost:

The total variable cost (TVC) was calculated according to the following formula:

TVC = rabbit value cost + total feed cost + total veterinary management cost + management cost(6)

where the rabbit value cost was the value of the rabbit at purchase and the management cost per rabbit was the cost for water, electricity, and labor in LE.

The total feed cost was determined as follows:

Total feed cost = cumulative feed consumption per rabbit × price per kg feed(7)

The total veterinary management cost (TVM) was calculated as follows:

TVM = disinfection cost + sanitation cost + treatment cost + vaccination cost(8)

where the disinfection cost was about 0.90 LE per rabbit, the sanitation cost was about 1.50 LE per rabbit, vaccination cost was about 4.00 LE per rabbit, and treatment cost represents the therapy used for the coccidial infections, including the cost of the herbal extracts, which was 5.00 LE per rabbit for PPE and no cost for the neem leaf extract.

Total fixed cost was estimated as the cost of building depreciation plus the cages. The depreciation period was 30 years for the building and 15 years for the cages [53].

Total cost was calculated by adding together the total fixed and variable costs [54,55].

Return parameters, including total return, net profit, capital turnover, and return on investment, were calculated according to the following formulas (Total return [56], Net profit [40], total cost [57], TC [58]):

Total return = average rabbit final weight × market price (45 LE for each kg)(9)

Net profit = total return − total cost(10)

Capital turnover (CTO) = TR ÷ TC(11)

Return on investment (ROI) = NP ÷ TC(12)

##### Relative Economic Efficiency Measures

The relative economic efficiency parameters (TR and NP) were calculated with regard to the positive and negative control groups as follows:

Relative TR = (TR of the tested group ÷ TR of the control group) × 100(13)

Relative NP = (NP of the tested group ÷ NP of the control group) × 100(14)

### 2.6. Statistical Analysis

The data were collected, arranged, summarized, and then analyzed statistically using the SPSS computer program [59] according to the following models:

Two-way analysis of variance (ANOVA) using a general linear model (GLM) that was constructed to determine the effect of the breed, treatment group, and breed × group interaction [60]. Significance was determined using Tukey’s test by the MSTAT program [61].

One-way ANOVA performed to determine the means of fold changes of different genes among different treatment groups and the anticoccidial index for each breed. Significance was determined using Tukey’s test.

Cross-tabulation analysis used for analysis of mortality percentage and livability percentage among the different treatment groups.

## 3. Results

### 3.1. Growth Performance

Changes in the growth performance parameters among the different experimental groups of NZ and VL rabbits before, during, and after coccidial infection are shown in Table 1 and Table 2. During the pre-infection period of the experiment, there were no significant (*p* > 0.05) differences in BW, FI, BWG, or FCR among the different treatment groups of the same rabbit breed.

In the 3rd week of the experimental period (during infection), herbal extract treatment groups (G3-N, G4-N, and G5-N) did not show any significant increase in BW compared to those in the positive control group (G2-N). However, the negative control groups (G1-N and G1-V) for both NZ and VL rabbits had a significantly higher BW and BWG when compared to positive control and herbal treatment groups within each breed (G2-N, G3-N, G4-N, and G5-N; G2-V, G3-V, G4-V, and G5-V). For FI, negative control groups (G1-N and G1-V) recorded the highest values, while the combined treatment groups (G5-N and G5-V) recorded the lowest values (non-significant values for NZ and significant values for VL). For FCR in the 3rd week (during infection), the positive control groups (G2-N and G2-V) recorded significantly (*p* ≤ 0.05) higher FCR (5.08 and 4.85 for NZ and VL breeds, respectively). Negative control groups (G1-N and G1-V) had significantly (*p* ≤ 0.05) lower FCR (2.41 and 2.65 for NZ and VL breeds, respectively) followed by the PPE treatment groups (G4-N and G4-V), with FCR values of 3.05 and 3.25 for NZ and VL breeds, respectively.

In the 5th and 7th weeks of the experimental period (2nd and 4th week post-infection), groups treated with either neem leaf extract or with the combined treatment (G3-N, G5-N, G3-V, and G5-V) showed a significant (*p* ≤ 0.05) increase in BW compared with the positive control groups (G2-N and G2-V) of the same breed. The negative control groups (G1-N and G1-V) for both NZ and VL breeds had BW higher than the other groups (G2-N, G3-N, G4-N, and G5-N; G2-V, G3-V, G4-V, and G5-V) within the same breed. While the positive control groups for both the NZ and VL breeds (G2-N and G2-V) recorded a significantly (*p* ≤ 0.05) lower cumulative BWG compared with other groups of the same breed, the difference in cumulative BWG among the different herbal treatment groups (G3-N, G4-N, and G5-N; G3-V, G4-V, and G5-V) within each breed was non-significant (*p* > 0.05).

In the 5th week of the experimental period, the VL breed PPE treatment group (G4-V) recorded the significantly (*p* ≤ 0.05) lowest value of FI, while the negative control groups (G1-N and G1-V) and combined treatment groups for both NZ and VL breeds (G5-N and G5-V) recorded significantly (*p* ≤ 0.05) higher FI values. The VL breed positive control group (G2-V) recorded the highest (significant, *p* ≤ 0.05) FCR, followed by the NZ breed positive control group (G1-N). The combined treatment groups for both breeds (G5-N and G5-V) and the VL breed negative control group (G1-V) recorded the lowest values of FCR. FCR values for VL breed treatment groups (G3-V, G4-V, and G5-V) were not significantly (*p* > 0.05) different from those of their negative control group (G1-V).

For FI in the 7th week of the experimental period, the NZ breed combined treatment group (G5-N) recorded a significantly lower FI than those in the other groups (G1-N, G2-N, G3-N, and G4-N) in the same breed. The VL breed negative control group (G1-V) recorded the highest FI values (1763.3 g, *p* ≤ 0.05). In both the NZ and VL breeds, there was a non-significant (*p* > 0.05) difference in FI between the negative control group (G1-N and G1-V) and the PPE treatment groups (G4-N and G4-V). For FCR in the 7th week of the experimental period, the NZ breed positive control group (G2-N) recorded the highest FCR (significant, *p* ≤ 0.05) and the VL breed neem leaf treatment group (G3-V) recorded the lowest FCR.

The cumulative FCR was significantly (*p* ≤ 0.05) higher in the positive control groups (G2-N and G2-V) compared with those in the negative control and herbal treatment groups (G1-N, G3-N, G4-N, and G5-N; G1-V, G3-V, G4-V, and G5-V) within the same breed. At the same time, there was a non-significant (*p* > 0.05) difference in cumulative FCR between the negative control (G1-N and G2-V) and herbal treatment groups (G3-N, G4-N, and G5-N; G3-V, G4-V, and G5-V) within the same breed. All the herbal treatment groups (G3-N, G4-N, G5-N, G3-V, G4-V, and G5-V) showed significantly (*p* ≤ 0.05) improved relative FCR compared to the positive control groups (G2-N and G2-V), which recorded the worst values.

With respect to the relative growth performance parameters, the herbal treatment groups (G3-N, G4-N, G5-N, 3G-V, G4-V, and G5-V) recorded a significantly higher relative BWG than rabbits in the positive control groups (G2-N and G2-V). There was a non-significant difference in relative BWG between the negative control group (G1-N) and the neem leaf treatment group (G3-N) for the NZ breed. The VL breed positive control group (G2-V) showed a greater loss in relative BWG than those of its NZ breed peers (G2-N).

Regarding differences between the breeds, the VL breed recorded significantly (*p* ≤ 0.05) higher BW and numerically higher BWG than the NZ breed during the experimental period. Daily gain and cumulative body weight gain were significantly (*p* ≤ 0.05) higher in the VL breed than in the NZ breed. FI for the VL breed was significantly (*p* ≤ 0.05) higher during the 7th week of the experiment (4th week post-infection) and had a numerically higher total FI than the NZ breed. The FCR in the 2nd and 7th weeks of the experimental period (FCR2 and FCR7) and the cumulative FCR were significantly (*p* ≤ 0.05) lower in the VL breed than in the NZ breed.

### 3.2. Parasitological Findings

Table 3 shows that the treatment with PPE, alone or combined with neem leaf extract, demonstrated satisfactory efficacy for fecal scoring from the first day of treatment to the end of the experiment through the alleviation of diarrhea and production of normal fecal pellets in both NZ and VL rabbit breeds. Moreover, the treatment with neem leaf extract in NZ rabbit breed groups (G-3N and G5-N), alone or in combination with PPE, showed a significant (*p* ≤ 0.05) reduction in the fecal score compared to those of the positive control group (G2-N) from the 6th to the 11th DPI, and a non-significant decrease on the 12th and 13th DPI. Fecal scoring in the VL breed neem leaf extract treatment group (G3-V) showed a non-significant difference in comparison to the positive control group (G-2V).

The effect of the different herbal extracts on oocyst shedding (OPG) of the intestinal *Eimeria* oocysts in the rabbits is shown in Table 4. Herbal extract treatment groups for both breeds (G3-N, G4-N, and G5-N; G3-V, G4-V, and G5-V) had a significant (*p* ≤ 0.05) reduction in mean values of OPG when compared to the positive control groups (G2-N and G2-V), respectively. Moreover, the lowest amount of oocyst shedding was recorded in the NZ breed combined treatment group (G5-N) from the 5th to the 21st DPI. The positive control groups (G2-N and G2-V) demonstrated a steady increase in the number of oocysts shed up to the 13th DPI, after which the oocyst numbers began to decrease.

### 3.3. Clinical and Pathological Changes

During daily observation after coccidial challenge, beginning on the 5th DPI, the rabbits in the positive control groups (G2-N and G2-V) showed clinical signs of coccidiosis with a loss of appetite and changes in fecal consistency, shape, and texture Figure S2. By the 8th DPI, the findings had become more pronounced, with observations of depression, distended abdomen, and profound diarrhea (watery feces occasionally tinged with blood). The infected rabbits were found to have dull, rough coats in addition to fecal staining around the perineum and on the extremities compared with the rabbits in the negative control groups (G1-N and G1-V), who were clean and showed no signs of disease. The rabbits in the herbal treatment groups (G3-N, G4-N, G4-V, G5-N, and G5-V) were apparently healthy, with good appetite and minor changes in fecal consistency. Their perineal areas and extremities were clean and dry compared with those of the rabbits in the positive control groups (G2-N and 2G-V). Interestingly, the V-line rabbits in the neem leaf extract treatment group (G3-V) suffered from diarrhea (watery consistency of feces) from the 7th to the 13th DPI in comparison with the apparently healthy NZ rabbits in the neem leaf extract treatment group (G3-N).

**Figure 1:**
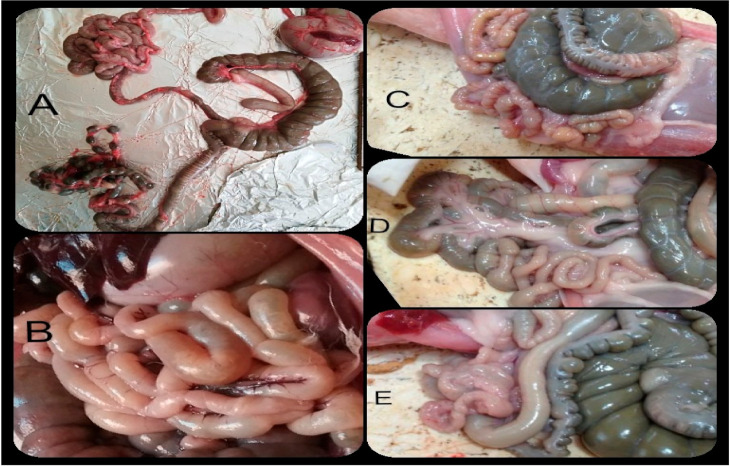
The macroscopic findings in the intestines in the experimental groups showing hyperemic wall, fluid contents, and multiple white patches on the mucosal surface with the highest score of 3 in the positive control group (**B**), a score of 2 in the pomegranate peel extract-treated group showing scattered white patches (**D**), a score of 1 in the neem leaf extract-treated group showing mild distention with grey green semisolid content (**C**), a score of 0 in the combined treated group (**E**), and in the negative control (**A**) apparently normal architecture. Data obtained from 3 rabbits/group.

On the 14th day post-coccidial infection, the macroscopic examination of the small intestine in the rabbits of the positive control groups (G2-N and G2-V) revealed severe congestion, distention, fluid contents, and multiple white patches on the mucosal surface of the small intestine. Tissue from these animals had significantly higher lesion scores (4.33 and 3.33 for G1-N and G1-V, respectively) compared to the apparently normal intestine of the negative control groups (G1-N and G1-V). While mild-to-moderate congestion with mild loose content and without gas distention was observed in the herbal treatment NZ and VL rabbits (G3-N, G4-N, and G5-N; G3-V, 4-V and G5-V), these rabbits still showed significantly reduced lesion scores for the small intestine compared with scores in the positive control groups (G2-N and G2-V). No coccidial infection lesions were recorded in the small intestine of the negative control groups (G1-N and G1-V) (Figure 1; Table 5). There were no gross pathological changes observed in the large intestine of all experimental groups for both the NZ and VL breeds, who presented with apparently healthy intestines that resulted in a score of zero. However, the prominent pathological findings that resulted in high scores in the cecum and colon were recorded only for the groups of VL rabbits treated with neem leaf extract alone or treated with the combined neem leaf extract and PPE (G3-V and G5-V) (Table 5).

**Figure 2:**
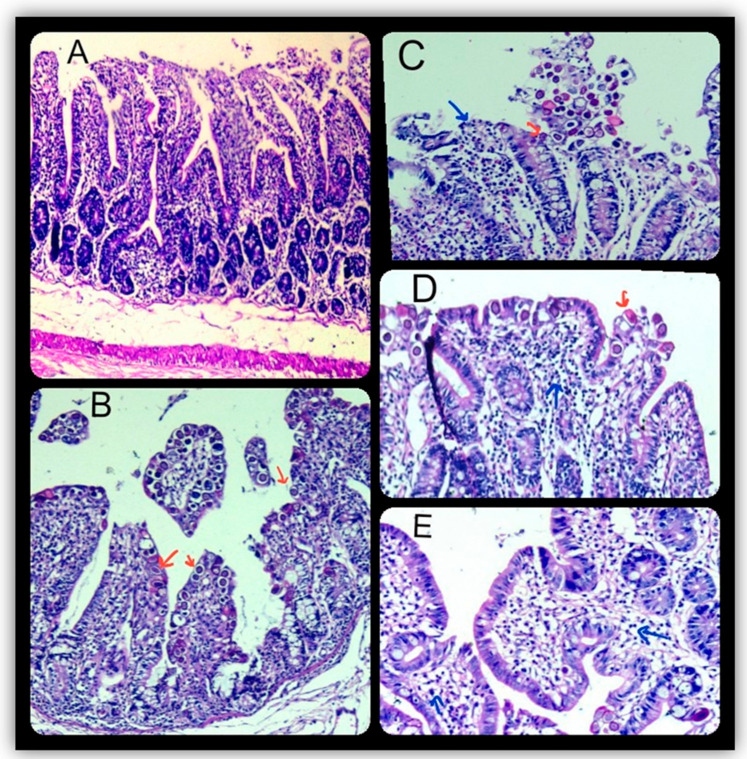
Microscopic findings in the intestines in the different experimental groups indicating: (**A**) apparently normal structure (HE × 100) in the negative control group, (**B**) positive control group showing sloughing and desquamation of the intestinal lining and different developmental stages of *Eimeria* spp. (red arrow) with lymphocytic infiltration (blue arrow) in the lamina propria and edema in the submucosa (HE × 100). (**C**–**E**), intestines from rabbits in the herbal treated groups showing a mild degree of sloughing of the epithelium lining and less infiltration with coccidial oocyte-infected epithelium (HE × 200). Data obtained from 3 rabbits/group. Blue arrow is pointing to lymphocytic infiltration in the epithelial lining while red arrow is pointing to developmental stages of *Eimeria* spp.

The histopathological data in Table 5 and Figure 2 show that post-mortem examination of the NZ and VL rabbits experimentally infected with coccidial oocysts (positive controls; G2-N and G2-V) revealed different developmental stages of *Eimeria* spp. The epithelial cells of the intestine that had been invaded by the parasites were found to have severe cell necrosis and desquamation of the epithelial lining at the lumen, which resulted in the highest significant scores compared with those of the negative control groups (G1-N and G1-V). Lymphocytic infiltration was also seen in the lamina propria. The intestines of the herbal treatment NZ and VL rabbits (G3-N, G4-N, and G5-N; G3-V, G4-V, and G5-V) showed marked intact epithelium lining, with few-to-none *Eimeria* spp. development stages and mild-to-marked lymphocytic infiltration in the lamina propria, which resulted in a significant decrease in the lesion scores when compared with those in the positive control groups (G2-N and G2-V). The intestine in the negative control groups showed normal architecture with the highest recorded score for the intact epithelium in comparison with the other infected groups. The histological analysis revealed that the average of the total HIS was the highest in the jejunum of the positive control groups of NZ and VL rabbits (G2-N and G2-V) and was significantly lower in the herbal treatment NZ and VL rabbits (G3-N, G4-N, and G5-N; G3-V, G4-V, and G5-V) compared to those in the positive control groups (G2-N and G2-V). The significant differences in the average total HIS between the groups that received neem leaf extract alone or combined treatment (G3-N and G5-N; G3-V and G5-V) and those that received the PPE treatment (G4-N and G4-V) in both the NZ and VL rabbits were smaller (Table 5).

The overall mortalities were 11.10% and 16.70% in the positive control groups of the NZ and VL rabbits (G2-N and G2-V), respectively. Post-mortem examination of the dead rabbits showed externally rough coats with feces-soiled perineum. Internal lesions included severe congestion, gas distention, and mucoid contents in different parts of the intestinal tract. Meanwhile, no mortalities were found in any of the other groups. No animals died in the negative control groups (G1-N and G1-V) and the relative level of protection in all the herbal treatment groups was 100% (G3-N, G3-V, G4-N, G4-V, G5-N, and G5-V) in both breeds, as shown in Table 6.

The values > 120 in the anticoccidial index (Table 6) imply that the NZ and VL rabbits infected with *Eimeria* species were susceptible to the herbal treatments with neem leaf extract and/or PPE. The ACI values between 120 and 160 in the infected groups 3-V, 4-V, and 5-V represented the low and partial efficacy of the neem leaf extract and/or PPE in the treatment of the VL rabbits infected with *Eimeria* species. Treatment of the NZ rabbits infected with *Eimeria* species with PPE (G4-N) showed a medium anticoccidial efficacy, with an ACI value between 160 and 180. The best and highest anticoccidial efficacy, with ACI values over 180, was recorded in the NZ rabbits treated with neem leaf extract alone or combined with PPE (G3-N and G5-N).

### 3.4. Immunological Parameters

**Figure 3:**
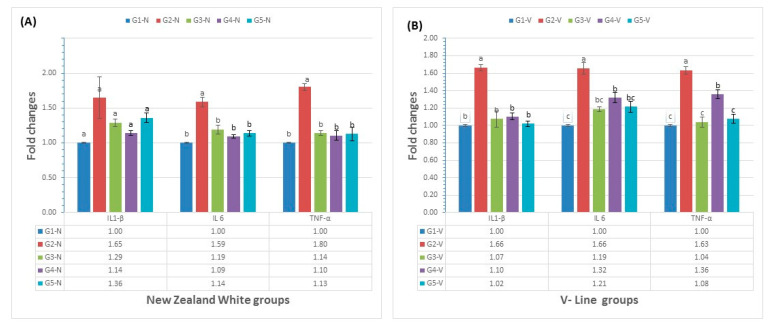
IL-1β, IL6, and TNF-α changes in NZ and VL rabbits in response to coccidial infection and herbal extract treatment. Data are presented as mean ± standard error of the mean. G1-N, negative control group of New Zealand rabbits; G2-N, positive control group of New Zealand rabbits; G3-N, neem leaf extract treatment of New Zealand rabbits; G4-N, pomegranate peel extract treatment of New Zealand rabbits; G5-N, combined treatment of New Zealand rabbits; G1-V, negative control group of V-line rabbits; G2-V, positive control group of V-line rabbits; G3-V, neem leaf extract treatment of V-line rabbits; G4-V, pomegranate peel extract treatment of V-line rabbits; G5-V, combined treatment of V-line rabbit. Data obtained from 3 rabbits/group. (**A**) New Zealand White groups. (**B**) V-Line groups. Means that have no superscript in common are significantly different from each other.

The mRNA levels of several cytokines (IL-1β, IL6, and TNF-α) were estimated in the jejunum of NZ and VL rabbits and their changes are shown in Figure 3. There was non-significant upregulation of IL-1β in the NZ breed positive control group (G2-N) compared to levels in the NZ breed negative control, neem leaf extract treatment, and PPE treatment groups (G1-N, G3-N, and G4-N). The VL breed positive control group (G2-V) demonstrated a significant (*p* ≤ 0.05) upregulation in mRNA levels of IL-1β compared to the VL breed negative control and herbal treatment groups (G1-V, G3-V, G4-V, and G5-V). The mRNA levels of the pro-inflammatory cytokines IL6 and TNF-α were significantly (*p* ≤ 0.05) upregulated in the positive control groups (G2-N and G2-V) compared with the negative control and herbal treatment groups of the same breed (G1-N, G3-N, G4-N, and G5-N; G1-V, G3-V, G4-V, and G5-V).

mRNA levels of IL6 and TNF-α did not significantly (*p* > 0.05) differ in the NZ breed negative control group (G1-N) compared with the challenged treatment groups. Additionally, in the VL breed, the negative control group (G1-V) showed a non-significant difference in mRNA levels of IL6 and TNF-α compared with the neem treatment or combined treatment groups (G3-V and G5-V). There was significant upregulation of the mRNA levels of IL6 and TNF-α in the VL breed PPE extract treatment group (G4-V) compared with the negative control group of the same breed (G1-V). There was downregulation of mRNA levels of IL-1β, IL6, and TNF-α in the herbal treatment groups compared with the levels of mRNA for these genes in the positive control groups of the same breed.

### 3.5. Carcass Traits

The effect of treatment with PPE and/or neem leaf extract on the carcass traits of NZ and VL rabbits experimentally infected with *Eimeria* spp. are shown in Table 7. The dressing, intestinal, stomach, and spleen percentages at the end of the experimental period showed non-significant (*p* > 0.05) differences between infected and non-infected groups within the same breed. The NZ breed negative control group (G1-N) recorded the highest dressing percentage and lowest intestinal percentage compared with other groups of both breeds (G2-N, G3-N, G4-N, and G5-N; G1-V, G2-V, G3-V, G4-V, and G5-V), with the highest intestinal percentage recorded in G2-V. The VL breed recorded a significantly (*p* ≤ 0.05) higher intestine percentage and significantly lower stomach and spleen percentage compared with the NZ breed.

### 3.6. Economic Efficiency

The effect of treatment with neem leaf extract and/or PPE on the economic efficiency measures of NZ and VL rabbits experimentally infected with *Eimeria* spp. are shown in Table 8. Economic efficiency measures include different costs (total veterinary management, feed cost, total variable cost, and total fixed cost), return parameters, and relative efficiency (relative return and relative net profit).

Regarding total veterinary management, the highest TVM was LE 11.40, recorded for the PPE treatment or combined treatment groups in both breeds (G4-N, G5-N, G4-V, and G5-V). The lowest TVM value (LE 6.40) was recorded for the rest of the groups (G1-N, G2-N, G3-N,1 G-V, G2-V, and G3-V). Concerning feed cost, the negative control groups for both NZ and VL breeds (G1-N and G1-V) recorded the highest feed cost values (significant value for VL and non-significant value for NZ breed) compared to the other positive control and herbal treatment groups (G2-N, G3-N, G4-N, G5-N, G2-V, G3-V, G4-V, and G5-V). The lowest feed cost, LE 22.95, was recorded for the NZ breed combined treatment group (G5-N). Regarding total variable cost (TVC) and total cost, the VL breed neem leaf extract treatment and the positive control groups (G3-V and G2-V) incurred significantly (*p* ≤ 0.05) lower TVC and TC compared with the negative control, PPE treatment, and combined treatment groups of the same breed (G1-V, G4-V, and G5-V). In the NZ breed, lower TVC and TC in the neem leaf extract treatment and positive control groups (G3-N and G2-N) were not significant. Additionally, the VL breed negative control group (G1-V) recorded the highest values (LE 74.47 and 77.72 for TVC and TC, respectively), while the lowest values were recorded for the VL breed positive control group (G2-V) (63.05 and 66.3 for TVC and TC, respectively). The herbal treatment groups for both the NZ and VL breeds (G3-N, G4-N, G5-N, G3-V, G4-V, and G5-V) showed significantly (*p* ≤ 0.05) higher TR and NP compared with the positive control groups of the same breed (G2-N and G2-V), which recorded the lowest values. The highest TR and NP values (significant, *p* ≤ 0.05) were recorded for the negative control groups of both breeds (G1-N and G1-V).

Capital turnover and return on investment showed significant (*p* ≤ 0.05) differences among the different groups, with the highest value recorded for the VL breed negative control group (G1-V) and the lowest value for the NZ breed positive control group (G2-N). TR and NP for the treatment groups (G3-N, G4-N, G5-N, G3-V, G4-V, and G5-V) increased compared to those of the positive control groups (G2-N and G2-V). Groups treated with either neem leaf extract and/or PPE exhibited less reduced TR and NP values in relation to negative control groups by more than twice the value of the positive control groups.

## 4. Discussion

At the end of the experimental period, *Eimeria*-infected NZ and VL rabbits in the positive control groups were found to be more affected by coccidial infection compared with those in the herbal-treated groups and negative control groups, as they had lower body weights and less body weight gain due to impaired FI and FCR. Similarly, Abdel-Haleem et al. and Metwaly et al. [62,63] demonstrated decreased body weight in *Eimeria*-infected rabbits compared with non-infected rabbits. Metwaly et al. [63] stated that the decrease in body weight occurred due to the damage to the FCR and was certainly also related to impaired enzymatic systems. Additionally, Dkhil [64] found that weight loss in infected mice was associated with watery, mucoid diarrhea and decreased intake of water and feed. Gres et al. [65] reported that *Eimeria* infections harmed growth and feed utilization, resulting in an imbalance of nutrition and interruption in digestion and absorption of feed [66]. In our study, use of either neem leaf extract and/or PPE resulted in improved growth performance with negligible BW losses in the herbal treatment groups for both breeds. Concomitant BW losses continued in the positive control groups for both breeds. Evidence of this improvement could be seen in the relative BWG and relative FCR for the herbal treatment groups, which were significantly higher than those of the positive control groups and not significantly different from those of the negative control groups. Neem (*Azadirachta indica*) is rich in azadiractoids, which possess anti-inflammatory activity [67]. Additionally, the anti-coccidial effect may be due to bioactive chemicals in neem, such as azadirachtin, which has a significant effect against parasites such as coccidial species [68,69]. Pomegranates also have antioxidative properties, as they are rich in anthocyanins, gallotannins, ellagitannins, gallagyl esters, hydroxybenzoic acids, hydroxycinnamic acids, and dihydroflavonol [70,71]. These compounds can be found in pomegranate leaves, which have been reported to produce anti-protozoal effects [72,73,74].

Our study used two different strategies for extraction (ethanolic extract for pomegranate peel and water extract for neem leaves). Studies in the literature used several different forms of extract, including ethanolic, methanolic, and water. Our results were in agreement with other published data, most of which showed the most beneficial health effects following treatment with ethanolic extracts [38,75,76]. In general, pomegranate peel extract has been confirmed as a highly valuable source of bioactive compounds that can be used to improve the functional characteristics of food when used in ethanolic form [76]. The mix of flavonoids, polyphenols, and other active ingredients found in pomegranate peel have been published previously, confirming that the antioxidant activity in the pomegranate can be attributed mainly to its polyphenol, quercetin, and rutin contents [77].

With respect to neem leaf extract prepared with water, for this plant, the water extract is more stable than other forms of extract [78,79,80]. The biological activity of neem extract can be attributed to the polyphenols it contains, as a recent study (2021) showed that rutin is the major phenolic component in neem extract [81]. Water extract of neem has been used to treat inflammation, cancer, cardiovascular disorders, heart attacks, and diabetes. Neem contains highly water-soluble phytochemicals (flavonoids, tannins, polyphenols, catechins, gallic acid, quercetins, and saponins) with more health-promoting and biological effects [82]. It is anticipated that neem leaf extract targeted at sites other than those used by antibiotics will be effective against drug-resistant pathogens [83].

The herbal extracts demonstrated a positive effect in the treatment of the infected NZ and VL rabbits by reducing the fecal score and the shedding of *Eimeria* oocysts compared to the positive control groups of both breeds, which was demonstrated clinically by the alleviation of diarrhea and production of normal fecal pellets. The anticoccidial efficacy of PPE is demonstrated by a reduction in the number of *Eimeria* oocysts in the feces of infected rabbits. This reduced output is due to chemical constituents in the pomegranate impairing *Eimeria* oocyst formation in the host, as described by Dkhil [64]. Negi et al. [71] explained that the main preventive action effected by pomegranates is to reduce oxidative damage in the rabbit gut caused by *Eimeria* infection. These findings were supported by Alzohairy [14], who stated that *Azadirachta indica* (neem) exhibits free radical scavenging activities due to abundant antioxidants as well as anti-inflammatory effects via regulation of pro-inflammatory enzyme activities. The anticoccidial activity of neem has been found to reduce the output of *E. papillata* oocysts in infected mice [68]. This reduced output indicates that neem prevents the parasite from developing in the intestinal cells before the inactive oocysts are formed and released. Despite having a high fecal score, the VL breed neem leaf extract treatment group had a lower oocyst count than the challenged untreated group.

In this study, the intestinal coccidiosis in the infected untreated NZ and VL rabbits led to observations of loss of appetite and changes in fecal consistency, shape, and texture on the 5th DPI, followed by more pronounced observations on the 8th DPI of depression, distended abdomen, and profound diarrhea (watery feces occasionally tinged with blood). The overall mortalities were 11.10% and 16.70% in the infected, untreated NZ and VL rabbits, respectively (Table 6). Post-mortem examination of the dead rabbits showed externally rough coats with feces-soiled perineums, and internal lesions included severe congestion, gas distention, mucoid contents, and multiple white patches of mucosa in different parts of the intestinal tract. Histologically, different developmental stages of *Eimeria* spp. invaded the epithelial cells of the intestine with severe cell necrosis, desquamation of the epithelial lining, and lymphocytic infiltration (Figure 2, Table 5).

These findings are in agreement with those recorded for naturally concurrent intestinal infections caused by more than one *Eimeria* species. Rabbits with these multiple coccidial infections suffered from severe diarrhea and their intestines appeared macroscopically congested with thickened, hemorrhagic, and ulcerated tissue, and microscopically sloughed and desquamated intestinal lining containing different developmental stages of *Eimeria* spp. They also exhibited severe lymphocyte infiltration in 67% of the NZ white rabbits examined in a study performed in Iran [84] and in 7.7%, 9.1%, and 48.5% of the Rex, English, and NZ rabbit breeds examined in a study performed in Egypt, respectively [85]. Moreover, the highest macroscopic and microscopic lesion scores and total HIS in the examined intestines from the untreated infected NZ and VL rabbits in our study were in agreement with the data recorded in the intestines of California white and NZ rabbit breeds as a result of artificially induced mixed *Eimeria* infections [10]. These findings are attributed to the pathogenicity and pathology caused by oral inoculation with *Eimeria* spp. sporulated oocysts, which results in oocyst excystment in the intestine followed by release of four sporocysts and eight sporozoites and the invasion of epithelial cells in different parts of the intestines by the sporozoites [6,86].

The results of herbal treatments with neem leaf extract and/or PPE, as shown in Table 5 and Table 6 and Figure 1 and Figure 2, had positive satisfactory effects on the NZ and VL rabbits experimentally infected with mixed *Eimeria* species, as evidenced by their healthy appearance, good appetite, no mortalities, and ACI over 120. Meanwhile, in all the herbal treatment groups for both breeds of rabbits, we observed a relative level of protection of 100%, significant reduction in the macroscopic intestinal lesion scores (mild-to-moderate congestion with mild loose content and without gas distention), the microscopic intestinal lesion scores (marked intact lining epithelium with few-to-none appearance of *Eimeria* development stages and mild-to-marked lymphocytic infiltration in the lamina propria), and the total HIS. These results agreed with data showing improved histopathological alterations, significant reduction of HIS, and a decreased number of *Eimeria* oocysts in the intestines of mice infected with *E*. *papillata* as the result of oral administration of neem and pomegranate extracts [64,68]. This may be due to the antiprotozoal activity of neem and pomegranate extracts and to potent antioxidant and anti-inflammatory activities that protect the host tissue from injuries caused by *Eimeria* oocysts [87]. The properties of neem and pomegranate extracts have previously been ascribed mainly to their major chemical compounds, azadirachtin [54] and anthocyanins [70,71], respectively. These compounds have been reported to produce anti-coccidial and antiprotozoal activity [72,88]. As natural products, plant (neem and/or pomegranate) extracts are promising sources for novel anticoccidial agents that target *Eimeria* and also have organ-protective properties that protect and heal the *Eimeria*-infected host tissues.

The use of the neem leaf extract in the treatment of the VL breed rabbits caused side effects from diarrhea and congestion of the large intestines with watery content when compared with the apparently healthy New Zealand white rabbits. This observation is supported by those of Jacobson and Schmutterer and Braga et al. [89,90], who reported mild diuretic properties accompanied by copious liquid filling and intestinal swelling in the guts of mammalian models, mainly mice, rats, and rabbits, due to the use of an aqueous extract of neem leaves. Other tissues and organs showed no obvious abnormalities.

In our study, there was upregulation of the mRNA levels of pro-inflammatory cytokines (IL-1β, IL6, and TNF-α) in both positive control groups compared with those of both negative control groups. Similarly, Abdel-Haleem et al. [62] reported upregulation of IL6 expression in *Eimeria*-infected rabbits compared with uninfected ones.

The results obtained in this study included downregulation of the mRNA levels of IL-1β, IL6, and TNF-α in the herbal treatment groups compared with the mRNA levels of these genes for both positive control groups, and a non-significant difference in mRNA levels of IL-1β, IL6, and TNF-α for both negative control groups and for the herbal treatment groups for both breeds. These findings demonstrate the anti-inflammatory effect of both pomegranate peel and neem leaf extracts. These findings partially concurred with those of Amer et al. [91], who reported that pomegranate reduced the mRNA levels of the pro-inflammatory cytokines IL-1β and TNF-α in *Eimeria*-infected mice treated with pomegranate compared to levels in untreated infected mice. Neem (*Azadirachta indica*) had an anti-inflammatory effect as it is rich in azadiractoids, which possess anti-inflammatory activity [67].

Regarding economic efficiency parameters, the herbal treatment groups for both breeds showed significantly higher TR and NP when compared with their respective positive control groups, which recorded the lowest values. Additionally, TR and NP for the NZ breed treated with herbal extracts showed a non-significant difference with those of their negative control group. In comparison, the VL breed treated with herbal extracts recorded significantly lower TR and NP than their negative control group. These results are due to improved growth performance in the groups treated with either neem, pomegranate, or a combination of both herbal extracts, which led to improved economic efficiency parameters (TR, NP) and relative efficiency measures compared to both positive control groups as well as a reduction in the number of rabbits lost to coccidial infection. These results are in agreement with those of several other studies [64,72,92], which reported the efficacy of pomegranate extract in the treatment of coccidiosis in mice and rabbits. They also agree with results that have reported that aqueous extract of neem showed a significant reduction in oocyst load without negatively affecting growth [93]. Neem has shown its potential as an inexpensive and natural anti-coccidial feedstuff [94]. We observed this in our study as well. The total cost increased in the PPE treatment and neem leaf extract + PPE treatment groups of NZ breed rabbits, as the treatment cost (pomegranate peel extract cost) was higher in these two specific groups than in any other treated groups.

## 5. Conclusions

It can be concluded that aqueous neem leaf extract and/or ethanolic pomegranate peel extract are possible safe therapeutic alternatives for use in the control of coccidiosis in rabbit production, as they have been shown to minimize the deleterious effects of intestinal coccidiosis in weaned rabbits due to their content of phytochemicals with anticoccidial properties. However, more in-depth research needs to be performed concerning their use in prophylaxis as natural alternatives to coccidiostats in animal feeds.

## Figures and Tables

**Table 1:** Bodyweight changes of the New Zealand white and V-line rabbits in response to coccidial infection and herbal extract treatment.

Variables		New Zealand White Rabbits						V-Line Rabbits						SEM	*p*-Value		
		G1-N	G2-N	G3-N	G4-N	G5-N	Overall	G1-V	G2-V	G3-V	G4-V	G5-V	Overall		B	G	B × G
Pre infection	BW1 (g)	606.00 ^b^	607.67 ^b^	607.00 ^b^	608.33 ^b^	605.67 ^b^	606.93 ^B^	705.33 ^a^	708.00 ^a^	705.00 ^a^	705.67 ^a^	706.00 ^a^	706.00 ^A^	0.84	**	NS	NS
	BW2 (g)	1056.80 ^b^	1051.80 ^b^	1054.50 ^b^	1050.00 ^b^	1052.50 ^b^	1053.10 ^B^	1178.90 ^a^	1177.20 ^a^	1179.20 ^a^	1173.40 ^a^	1175.80 ^a^	1176.90 ^A^	7.64	**	NS	NS
During infection	BW3 (g	1294.20 ^b,c,d^	1163.50 ^f^	1206.20 ^e,f^	1224.20 ^d,e,f^	1197.50 ^e,f^	1217.10 ^B^	1516.80 ^a^	1322.60 ^b,c^	1316.20 ^b,c^	1361.70 ^b^	1259.30 ^c,d,e^	1355.30 ^A^	8.41	**	**	NS
Post Infection	BW5 (g)	1650.60 ^b,c^	1400.20 ^f^	1549.40 ^c,d,e^	1495.00 ^e,f^	1637.30 ^b,c,d^	1546.50 ^B^	2037.00 ^a^	1522.00 ^d,e^	1651.20 ^b,c^	1667.60 ^b,c^	1750.00 ^b^	1725.60 ^A^	13.91	**	**	*
	BW7 (g)	1949.20 ^c,d^	1603.50 ^f^	1839.80 ^d,e^	1824.20 ^d,e^	1817.30 ^d,e^	1806.80 ^B^	2508.70 ^a^	1793.10 ^e^	2104.60 ^b^	2054.20 ^b,c^	2085.80 ^b,c^	2109.30 ^A^	16.22	**	**	**
Pre infection	BWG2 (g)	450.83 ^a^	444.17 ^a^	447.50 ^a^	441.67 ^a^	446.88 ^a^	446.21 ^A^	473.61 ^a^	469.17 ^a^	474.25 ^a^	467.72 ^a^	469.83 ^a^	470.92 ^A^	7.22	NS	NS	NS
During infection	BWG3 (g)	237.36 ^b^	111.67 ^d,e^	151.67 ^c,d^	174.17 ^c,d^	144.96 ^c,d,e^	163.96 ^A^	337.83 ^a^	145.42 ^c,d,e^	137.00 ^c,d,e^	188.34 ^b,c^	83.50 ^e^	178.42 ^A^	7.24	NS	**	*
Post Infection	BWG5 (g)	356.39 ^b,c^	236.67 ^c,d^	343.19 ^b,c^	270.83 ^c,d^	439.84 ^a,b^	329.38 ^A^	520.22 ^a^	199.44 ^d^	335.00 ^b,c^	305.83 ^c,d^	490.67 ^a^	370.23 ^A^	15.11	NS	**	NS
	BWG7 (g)	298.67 ^b,c,d^	203.33 ^d,e^	290.45 ^b,c,d^	329.17 ^b,c^	180.00 ^e^	260.32 ^B^	471.67 ^a^	271.11 ^c,d,e^	453.33 ^a^	386.67 ^a,b^	335.83 ^b,c^	383.72 ^A^	11.45	**	**	NS
Daily gain (g)		27.41 ^b,c^	20.32 ^e^	25.16 ^c^	24.81 ^c,d^	24.73 ^c,d^	24.49 ^B^	36.80 ^a^	22.15 ^d,e^	28.56 ^b^	27.52 ^b,c^	28.16 ^b^	28.64 ^A^	0.33	**	**	**
Cumulative BWG (g)		1343.20 ^b,c^	995.83 ^e^	1232.80 ^c^	1215.80 ^c,d^	1211.70 ^c,d^	1199.90 ^B^	1803.30 ^a^	1085.10 ^d,e^	1399.60 ^b^	1348.60 ^b,c^	1379.80 ^b^	1403.30 ^A^	15.96	**	**	**

BW1, initial body weight; BW2, body weight at 2nd week of experiment (pre-infection); BW3, body weight at 3rd week of experiment (during infection); BW5, body weight at 5th week of experiment (2nd week post-infection); BW7, body weight at 7th week of experiment (4th week post-infection); BWG2, body weight gain from start of experiment to 2nd week (pre-infection); BWG3, body weight gain from 2nd week to 3rd week of experiment (during infection); BWG5: body weight gain from 3rd week to 5th week of experiment (2nd week post-infection); BWG7, body weight gain from 5th week to 7th week of experiment (4th week post-infection). B, breed effect; G, group effect; B × G, breed and group interaction; * *p* ≤ 0.05, ** *p* ≤ 0.01, NS (non-significant): *p* > 0.05. G1-N, negative control group of New Zealand rabbits; G2-N, positive control group of New Zealand rabbits; G3-N, neem leaf extract treatment of New Zealand rabbits; G4-N, pomegranate peel extract treatment of New Zealand rabbits; G5-N, combined treatment of New Zealand rabbits; G1-V, negative control group of V-line rabbits; G2-V, positive control group of V-line rabbits; G3-V, neem leaf extract treatment of V-line rabbits; G4-V, pomegranate peel extract treatment of V-line rabbits; G5-V, combined treatment of V-line rabbits. Means carrying ^a–f^ significantly differ among different groups of the same row, while means carrying ^A,B^ significantly differ among New Zealand and V-line of the same row.

**Table 2:** Feed intake and feed conversion rate changes of the New Zealand white and V-line rabbits in response to coccidial infection and herbal extract treatment.

Variables		New Zealand White Rabbits						V-Line Rabbits						SEM	*p*-Value		
		G1-N	G2-N	G3-N	G4-N	G5-N	Overall	G1-V	G2-V	G3-V	G4-V	G5-V	Overall		B	G	B × G
Pre infection	FI2 (g)	1312.50 ^a,b^	1375.80 ^a^	1337.10 ^a,b^	1250.80 ^a,b^	1239.40 ^a,b^	1303.10 ^A^	1286.20 ^a,b^	1258.30 ^a,b^	1291.50 ^a,b^	1163.30 ^b^	1182.50 ^b^	1236.40 ^A^	21.28	NS	NS	NS
During infection	FI3 (g)	570.56 ^b,c^	563.33 ^b,c^	537.92 ^b,c^	526.94 ^c^	525.00 ^c^	544.75 ^B^	896.67 ^a^	705.00 ^b^	597.92 ^b,c^	603.33 ^b,c^	342.00 ^d^	628.98 ^A^	20.13	*	**	*
Post infection	FI5 (g)	1543.30 ^a,b^	1159.40 ^c,d^	1187.80 ^c,d^	1210.30 ^c,d^	1390.40 ^a,b,c^	1298.20 ^A^	1612.50 ^a^	1075.80 ^c,d^	1261.70 ^b,c,d^	993.33 ^d^	1365.80 ^a,b,c^	1261.80 ^A^	37.88	NS	**	NS
	FI7 (g)	1425.80 ^b^	1291.70 ^b,c^	1408.30 ^b^	1300.80 ^b,c^	1018.50 ^c^	1289.00 ^B^	1763.30 ^a^	1309.40 ^b,c^	1385.00 ^b^	1601.70 ^a,b^	1476.70 ^a,b^	1507.20 ^A^	37.23	**	*	NS
Total feed consumption (g)		4852.20 ^b^	4390.30 ^b,c^	4471.10 ^b,c^	4288.90 ^b,c^	4173.30 ^c^	4435.20 ^A^	5558.80 ^a^	4348.60 ^b,c^	4536.10 ^b,c^	4361.70 ^b,c^	4367.00 ^b,c^	4634.40 ^A^	71.69	NS	**	NS
Pre infection	FCR2	2.94 ^a,b^	3.11 ^a^	2.98 ^a,b^	2.83 ^a,b^	2.77 ^a,b^	2.93 ^A^	2.77 ^a,b^	2.70 ^a,b^	2.72 ^a,b^	2.49 ^b^	2.52 ^b^	2.64 ^B^	0.06	*	NS	NS
During infection	FCR3	2.41 ^e^	5.08 ^a^	3.58 ^b,c,d^	3.05 ^c,d,e^	3.63 ^b,c,d^	3.55 ^A^	2.65 ^d,e^	4.85 ^a^	4.41 ^a,b^	3.25 ^c,d,e^	4.09 ^a,b,c^	3.85 ^A^	0.13	NS	**	NS
Post infection	FCR5	4.33 ^a,b,c,d^	4.92 ^a,b^	3.51 ^c,d,e^	4.47 ^a,b,c^	3.15 ^e^	4.08 ^A^	3.08 ^e^	5.42 ^a^	3.77 ^b,c,d,e^	3.25 ^c,d,e^	2.83 ^e^	3.67 ^A^	0.19	NS	*	NS
	FCR7	4.77 ^b,c^	6.33 ^a^	4.85 ^b,c^	3.96 ^c,d^	5.66 ^a,b^	5.12 ^A^	3.76 ^c,d^	4.83 ^b,c^	3.10 ^d^	4.14 ^c,d^	4.67 ^b,c^	4.10 ^B^	0.14	**	**	NS
Cumulative FCR		3.62 ^b,c^	4.41 ^a^	3.62 ^b,c^	3.53 ^b,c^	3.44 ^c^	3.73 ^A^	3.08 ^c^	4.04 ^a,b^	3.27 ^c^	3.23 ^c^	3.18 ^c^	3.36 ^B^	0.06	**	**	NS
BWG% to control negative%		100.00 ^a^	74.14 ^c^	91.78 ^a,b^	90.51 ^b^	90.20 ^b^	89.33 ^A^	100.00 ^a^	60.17 ^d^	77.61 ^c^	74.78 ^c^	76.52 ^c^	77.82 ^B^	0.96	**	**	NS
BWG% to control positive		134.89 ^b^	100.00 ^d^	123.80 ^b,c^	122.09 ^b,c^	121.67 ^c^	120.49 ^B^	166.19 ^a^	100.00^d^	128.98 ^b,c^	124.28 ^b,c^	127.16 ^b,c^	129.32 ^A^	1.50	**	**	*
FCR% to control negative		100.01 ^c^	121.94 ^a,b^	100.19 ^c^	97.60 ^c^	95.17 ^c^	102.98 ^A^	100.03 ^c^	131.38 ^a^	106.26 ^b,c^	104.86 ^b,c^	103.19 ^c^	109.14 ^A^	1.96	NS	**	NS
FCR% to control positive		82.05 ^b^	100.04 ^a^	82.19 ^b^	80.07 ^b^	78.07 ^b^	84.94 ^A^	76.19 ^b^	100.07 ^a^	80.93 ^b^	79.86 ^b^	78.59 ^b^	83.13 ^A^	1.60	NS	**	NS

FI2, feed intake from 0 to 2nd week of experiment (pre-infection); FI3, feed intake from 2nd to 3rd week of experiment (during infection); FI5, feed intake from 3rd to 5th week of experiment (2nd week post-infection); FI7, feed intake from 5th to 7th week of experiment (4th week post-infection); FCR2, feed conversion rate from 0 to 2nd week of experiment (pre-infection); FCR3, feed conversion rate from 2nd to 3rd week of experiment (during infection); FCR5, feed conversion rate from 3rd to 5th week of experiment (2nd week post infection); FCR7, feed conversion rate from 5th to 7th week of experiment (4th week post-infection). B, breed effect; G, group effect; B × G, breed and group interaction; * *p* ≤ 0.05, ** *p* ≤ 0.01, NS (non-significant): *p* > 0.05. G1-N, negative control group of New Zealand rabbits; G2-N, positive control group of New Zealand rabbits; G3-N, neem leaf extract treatment of New Zealand rabbits; G4-N, pomegranate peel extract treatment of New Zealand rabbits; G5-N, combined treatment of New Zealand rabbits; G1-V, negative control group of V-line rabbits; G2-V, positive control group of V-line rabbits; G3-V, neem leaf extract treatment of V-line rabbits; G4-V, pomegranate peel extract treatment of V-line rabbits; G5-V, combined treatment of V-line rabbits. Means carrying ^a–e^ significantly differ among different groups of the same row, while means carrying ^A,B^ significantly differ among New Zealand and V-line of the same row.

**Table 3:** Fecal score of the New Zealand white and V-line rabbits in response to coccidial infection and herbal extract treatment.

Variables	New Zealand White Rabbits						V-Line Rabbits						SEM	*p*-Value		
	G1-N	G2-N	G3-N	G4-N	G5-N	Overall	G1-V	G2-V	G3-V	G4-V	G5-V	Overall		B	G	B × G
Day 5	0.00 ^d^	3.00 ^b^	2.00 ^b,c^	2.00 ^b,c^	1.67 ^b,c^	1.73	0.00 ^d^	4.67 ^a^	0.67 ^c,d^	2.67 ^b^	2.00 ^b,c^	2.00	0.19	NS	**	NS
Day 6	0.00 ^d^	4.00 ^a^	1.00 ^b,c,d^	0.67 ^c,d^	1.67 ^b,c,d^	1.47	0.00 ^d^	4.33 ^a^	2.00 ^b,c^	2.67 ^a,b^	1.33 ^b,c,d^	2.07	0.21	NS	**	NS
Day 7	0.00 ^d^	5.33 ^a^	0.67 ^c,d^	1.00 ^b,c,d^	3.00 ^a,b,c^	2.00	0.00 ^d^	4.33 ^a^	4.00 ^a^	3.33 ^a,b^	0.00 ^d^	2.33	0.29	NS	**	*
Day 8	0.00 ^e^	7.00 ^a^	1.00 ^d,e^	2.33 ^c,d,e^	3.67 ^b,c,d^	2.80	0.00 ^e^	6.33 ^a,b^	5.33 ^a,b,c^	1.33 ^d,e^	0.67 ^d,e^	2.73	0.37	NS	**	NS
Day 9	0.00 ^e^	7.00 ^a^	2.00 ^c,d,e^	1.33 ^d,e^	3.00 ^b,c,d^	2.67	0.00 ^e^	4.00 ^b,c^	4.67 ^b^	1.33 ^d,e^	1.67 ^d,e^	2.33	0.24	NS	**	*
Day 10	0.00 ^d^	6.00 ^a^	2.00 ^c^	1.33 ^c,d^	1.00 ^c,d^	2.07	0.00 ^d^	4.00 ^b^	4.67 ^a,b^	2.00 ^c^	2.00 ^c^	2.53	0.21	NS	**	*
Day 11	0.00 ^b^	4.00 ^a^	1.33 ^b^	0.00 ^b^	0.33 ^b^	1.13 ^A^	0.00 ^b^	4.00 ^a^	4.33 ^a^	0.00 ^b^	1.33 ^b^	1.93 ^A^	0.23	NS	**	NS
Day 12	0.00 ^c^	2.33 ^b^	1.33 ^b,c^	0.00 ^c^	0.00 ^c^	0.73 ^B^	0.00 ^c^	2.33 ^b^	5.00 ^a^	0.00 ^c^	1.33 ^b,c^	1.73 ^A^	0.19	*	**	*
Day 13	0.00 ^c^	1.33 ^b^	0.67 ^b,c^	0.00 ^c^	0.00 ^c^	0.40 ^B^	0.00 ^c^	0.67 ^b,c^	4.00 ^a^	0.00 ^c^	1.33 ^b^	1.20 ^A^	0.15	**	**	**
Day 14	0.00 ^b^	1.67 ^a^	0.00 ^b^	0.00 ^b^	0.33 ^a,b^	0.40 ^B^	0.00 ^b^	0.67 ^a,b^	1.67 ^a^	0.00 ^b^	1.00 ^a,b^	0.67 ^A^	0.18	NS	*	NS

B, breed effect; G, group effect; B × G, breed and group interaction; * *p* ≤ 0.05, ** *p* ≤ 0.01, NS (non-significant): *p* > 0.05. G1-N, negative control group of New Zealand rabbits; G2-N, positive control group of New Zealand rabbits; G3-N, neem leaf extract treatment of New Zealand rabbits; G4-N, pomegranate peel extract treatment of New Zealand rabbits; G5-N, combined treatment of New Zealand rabbits; G1-V, negative control group of V-line rabbits; G2-V, positive control group of V-line rabbits; G3-V, neem leaf extract treatment of V-line rabbits; G4-V, pomegranate peel extract treatment of V-line rabbits; G5-V, combined treatment of V-line rabbits. Means carrying ^a–e^ significantly differ among different groups of the same row, while means carrying ^A,B^ significantly differ among New Zealand and V-line of the same row.

**Table 4:** Oocyst count per gram of feces (OPG) of the New Zealand white and V-line rabbits in response to coccidial infection and herbal extract treatment.

Variables	New Zealand White Rabbits						V-Line Rabbits						SEM		*p*-Value	
	G1-N	G2-N	G3-N	G4-N	G5-N	Overall	G1-V	G2-V	G3-V	G4-V	G5-V	Overall		B	G	B × G
Day 5	0.00 ^g^	18.50 ^a^	0.60 ^f,g^	3.33 ^e^	1.27 ^f^	4.74 ^B^	0.00 ^g^	10.00 ^b^	7.07 ^c,d^	8.00 ^c^	6.63 ^d^	6.34 ^A^	0.13	**	**	**
Day 7	0.00 ^f^	24.73 ^c^	13.27 ^d,e^	14.37 ^d^	12.57 ^e^	12.99 ^B^	0.00 ^f^	59.67 ^a^	24.33 ^c^	32.67 ^b^	23.20 ^c^	27.97 ^A^	0.18	**	**	**
Day 9	0.00 ^g^	49.87 ^b^	31.20 ^e,f^	32.27 ^e^	29.60 ^f^	28.59 ^B^	0.00 ^g^	63.33 ^a^	36.40 ^d^	46.53 ^c^	31.33 ^e,f^	35.52 ^A^	0.20	**	**	**
Day 11	0.00 ^h^	91.47 ^a^	38.93 ^f^	45.47 ^d^	32.00 ^g^	41.57 ^A^	0.00 ^h^	82.27 ^b^	42.13 ^e^	48.53 ^c^	34.20 ^g^	41.43 ^A^	0.33	NS	**	**
Day 13	0.00 ^g^	273.03 ^a^	102.13 ^e^	151.60 ^d^	84.00 ^f^	122.15 ^A^	0.00 ^g^	248.53 ^b^	98.00 ^e^	166.67 ^c^	81.87 ^f^	119.01 ^B^	0.74	*	**	**
Day 15	0.00 ^i^	129.87 ^b^	29.87 ^g^	36.93 ^f^	25.33 ^h^	44.40 ^B^	0.00 ^i^	190.40 ^a^	77.27 ^d^	120.00 ^c^	63.53 ^e^	90.24 ^A^	0.38	**	**	**
Day 17	0.00 ^g^	21.07 ^d^	4.47 ^f^	9.33 ^e^	3.00 ^f^	7.57 ^B^	0.00 ^g^	47.47 ^a^	26.60 ^c^	30.80 ^b^	22.47 ^d^	25.47 ^A^	0.25	**	**	**
Day 19	0.00 ^e^	1.50 ^b^	0.37 ^d^	0.60 ^d^	0.30 ^d,e^	0.55 ^B^	0.00 ^e^	2.20 ^a^	1.00 ^c^	1.40 ^b^	0.40 ^d^	1.00 ^A^	0.035	**	**	**
Day 21	0.00 ^d^	0.93 ^b^	0.16 ^d^	0.40 ^c^	0.17 ^d^	0.33 ^B^	0.00 ^d^	2.00 ^a^	0.13 ^d^	0.73 ^b^	0.07 ^d^	0.59 ^A^	0.025	**	**	**

B, breed effect; G, group effect; B × G, breed and group interaction; * *p* ≤ 0.05, ** *p* ≤ 0.01, NS (non-significant): *p* > 0.05. G1-N, negative control group of New Zealand rabbits; G2-N, positive control group of New Zealand rabbits; G3-N, neem leaf extract treatment of New Zealand rabbits; G4-N, pomegranate peel extract treatment of New Zealand rabbits; G5-N, combined treatment of New Zealand rabbits; G1-V, negative control group of V-line rabbits; G2-V, positive control group of V-line rabbits; G3-V, neem leaf extract treatment of V-line rabbits; G4-V, pomegranate peel extract treatment of V-line rabbits; G5-V, combined treatment of V-line rabbits. Means carrying ^a–i^ significantly differ among different groups of the same row, while means carrying ^A,B^ significantly differ among New Zealand and V-line of the same row.

**Table 5:** Pathological lesion scoring in the intestines of the New Zealand white and V-line rabbits in response to coccidial infection and herbal extract treatment.

Variables		New Zealand White Rabbits						V-Line Rabbits						SEM	*p*-Value		
		G1-N	G2-N	G3-N	G4-N	G5-N	Overall	G1-V	G2-V	G3-V	G4-V	G5-V	Overall		B	G	B × G
**Macroscopic lesion scoring #**	Duodenum	0	0	0	0	0	0	0	0	0	0	0	0	-	-	-	-
	Jejunum	0.00 ^c^	2.67 ^a^	0.00 ^c^	0.33 ^c^	1.33 ^b^	0.87 ^A^	0.00 ^c^	2.67 ^a^	1.33 ^b^	1.00 ^b^	1.00 ^b^	1.20 ^A^	0.07	*	**	*
	Ileum	0.00 ^d^	0.67 ^b,c,d^	0.33 ^c,d^	1.00 ^a,b,c^	0.67 ^b,c,d^	0.53 ^A^	0.00 ^d^	1.67 ^a^	1.33 ^a,b^	0.67 ^b,c,d^	1.33 ^a,b^	1.00 ^A^	0.09	*	**	NS
	Small intestine	0.00 ^f^	3.33 ^b^	0.33 ^f^	1.33 ^e^	2.00 ^c,d,e^	1.40 ^A^	0.00 ^f^	4.33 ^a^	2.67 ^b,c^	1.67 ^d,e^	2.33 ^c,d^	2.20 ^A^	0.10	**	**	*
	Cecum	0	0	0	0	0	0.00 ^B^	0	0	3	0	2	1.00 ^A^	-	-	-	-
	Colon	0.00 ^c^	0.00 ^c^	0.00 ^c^	0.00 ^c^	0.00 ^c^	0.00 ^B^	0.00 ^c^	0.00 ^c^	2.00 ^a^	0.00 ^c^	1.67 ^b^	0.73 ^A^	0.03	**	**	**
	Large intestine	0.00 ^c^	0.00 ^c^	0.00 ^c^	0.00 ^c^	0.00 ^c^	0.00 ^B^	0.00 ^c^	0.00 ^c^	5.00 ^a^	0.00 ^c^	3.67 ^b^	1.73 ^A^	0.03	**	**	**
**Microscopic lesion scoring ##**	Intact epithelium	4.00 ^a^	0.67 ^e^	3.33 ^a,b,c^	2.67 ^c^	3.67 ^a,b^	2.87 ^A^	4.00 ^a^	1.00 ^d,e^	1.33 ^d,e^	3.00 ^b,c^	1.67 ^d^	2.20 ^A^	0.08	**	**	**
	Desquamated epithelium	0.00 ^f^	3.33 ^b^	0.00 ^f^	1.33 ^c^	0.33 ^e^	1.00 ^A^	0.00 ^f^	3.67 ^a^	3.33 ^b^	0.67 ^d^	1.33 ^c^	1.80 ^A^	0.11	**	**	**
	lymphocytic Infiltration	0	1	1	3	1	1.20 ^A^	0	2	0	2	0	0.80 ^A^	-	-	-	-
	Infected epithelium	0.00 ^f^	3.67 ^a^	3.00 ^a,b^	3.33 ^a^	2.33 ^b,c^	2.47 ^A^	0.00 ^f^	3.67 ^a^	1.00 ^d,e^	1.67 ^c,d^	0.67 ^e,f^	1.40 ^B^	0.08	**	**	**
	HIS	4.00 ^d^	11.00 ^a^	6.33 ^c^	8.67 ^b^	6.67 ^c^	7.33 ^A^	4.00 ^d^	12.00 ^a^	6.00 ^c^	9.00 ^b^	5.00 ^c,d^	7.20 ^A^	0.20.	NS	**	**

**#** Macroscopic lesion in jejunum scoring according to [45]: 0 with no evident lesion, while a score of 3 was assigned to severely infected rabbits. **##** Histopathological lesions in jejunum section were detected using a light microscope and scored from 0 to 4 in the different groups according to the nature and extent of the infection [10]. 0, Minimal (<10%); 1, Mild (11–20%); 2, Moderate (21–40%); 3, Marked (41–100%). HIS (total histological injury score). B, breed effect; G, group effect; B × G, breed and group interaction; * *p* ≤ 0.05, ** *p* ≤ 0.01, NS (non-significant): *p* > 0.05. G1-N, negative control group of New Zealand rabbits; G2-N, positive control group of New Zealand rabbits; G3-N, neem leaf extract treatment of New Zealand rabbits; G4-N, pomegranate peel extract treatment of New Zealand rabbits; G5-N, combined treatment of New Zealand rabbits; G1-V, negative control group of V-line rabbits; G2-V, positive control group of V-line rabbits; G3-V, neem leaf extract treatment of V-line rabbits; G4-V, pomegranate peel extract treatment of V-line rabbits; G5-V, combined treatment of V-line rabbits. Data obtained from 3 rabbits/group. Means carrying ^a–f^ significantly differ among different groups of the same row, while means carrying ^A,B^ significantly differ among New Zealand and V-line of the same row.

**Table 6:** Livability (%), mortality (%), relative level of protection (RLP; %), and anticoccidial index [48] of the New Zealand white and V-line rabbits in response to coccidial infection and herbal extract treatment.

Breed	Groups	Livability (%)	Mortality (%)	RLP (%)	ACI
New Zealand White Rabbits	G1-N	100%	0.00%	100%	235.23 ^a^± 5.12
	G2-N	88.90%	11.10%	0.00%	55.57 ^c^± 4.80
	G3-N	100%	0.00%	100%	200.79 ^b^± 2.74
	G4-N	100%	0.00%	100%	176.66 ^b^± 8.69
	G5-N	100%	0.00%	100%	191.74 ^b^± 9.47
V-Line Rabbits	G1-V	100.00%	0.00%	100%	251.69 ^a^± 11.52
	G2-V	83.30%	16.70%	0.00%	39.97 ^c^± 4.36
	G3-V	100.00%	0.00%	100%	139.35 ^b^± 6.91
	G4-V	100.00%	0.00%	100%	140.05 ^b^± 3.63
	G5-V	100.00%	0.00%	100%	154.37 ^b^± 6.03
*p*-value		**	**		**

G1-N, negative control group of New Zealand rabbits; G2-N, positive control group of New Zealand rabbits; G3-N, neem leaf extract treatment of New Zealand rabbits; G4-N, pomegranate peel extract treatment of New Zealand rabbits; G5-N, combined treatment of New Zealand rabbits; G1-V, negative control group of V-line rabbits; G2-V, positive control group of V-line rabbits; G3-V, neem leaf extract treatment of V-line rabbits; G4-V, pomegranate peel extract treatment of V-line rabbits; G5-V, combined treatment of V-line rabbits. ACI values with different superscripts ^a–c^ within the same row were significantly different at ** *p* ≤ 0.01 among different treatment groups of the same breed.

**Table 7:** Carcass changes of the New Zealand white and V-line rabbits in response to coccidial infection and herbal extract treatment.

Variables	New Zealand White Rabbits						V-Line Rabbits						SEM	*p*-Value		
	G1-N	G2-N	G3-N	G4-N	G5-N	Overall	G1-V	G2-V	G3-V	G4-V	G5-V	Overall		B	G	B × G
Dressing%	59.90 ^a^	56.51 ^a,b^	56.30 ^a,b^	57.16 ^a,b^	56.49 ^a,b^	57.27 ^A^	56.00 ^a,b^	57.03 ^a,b^	54.06 ^b^	56.83 ^a,b^	57.53 ^a,b^	56.29 ^A^	0.65	NS	NS	NS
Intestine%	8.95 ^d^	9.86 ^c,d^	10.95 ^c,d^	11.37 ^b,c,d^	11.17 ^c,d^	10.46 ^B^	12.27 ^a,b,c^	14.42 ^a^	11.95 ^a,b,c^	14.23 ^a,b^	11.49 ^a,b,c,d^	12.87 ^A^	0.34	**	NS	NS
Stomach%	4.67 ^b^	4.36 ^a,b^	4.50 ^a,b^	6.13 ^a^	5.61 ^a,b^	5.06 ^A^	3.92 ^b^	4.19 ^b^	3.97 ^b^	4.46 ^a,b^	4.63 ^a,b^	4.23 ^B^	0.21	*	NS	NS
Liver%	3.04 ^b,c^	2.67 ^c^	3.40 ^a,b^	3.35 ^a,b^	3.44 ^a,b^	3.18 ^A^	3.79 ^a^	3.95 ^a^	3.56 ^a,b^	3.08 ^b,c^	3.01 ^b,c^	3.48 ^A^	0.07	NS	NS	**
Spleen%	0.18 ^a,b^	0.16 ^a,b,c^	0.23 ^a^	0.16 ^a,b,c^	0.16 ^a,b,c^	0.18 ^A^	0.11 ^b,c^	0.09 ^c^	0.17 ^a,b,c^	0.15 ^b,c^	0.14 ^b,c^	0.13 ^B^	0.008	**	NS	NS
Kidney%	1.17 ^a^	0.95 ^a,b^	0.83 ^b,c^	0.79 ^b,c^	0.95 ^a,b^	0.94 ^A^	0.79 ^b,c^	0.74 ^b,c^	0.78 ^b,c^	0.88 ^b,c^	0.68 ^c^	0.77 ^B^	0.02	**	NS	NS

B, breed effect; G, group effect; B × G, breed and group interaction; * *p* ≤ 0.05, ** *p* ≤ 0.01, NS (non-significant): *p* > 0.05. G1-N, negative control group of New Zealand rabbits; G2-N, positive control group of New Zealand rabbits; G3-N, neem leaf extract treatment of New Zealand rabbits; G4-N, pomegranate peel extract treatment of New Zealand rabbits; G5-N, combined treatment of New Zealand rabbits; G1-V, negative control group of V-line rabbits; G2-V, positive control group of V-line rabbits; G3-V, neem leaf extract treatment of V-line rabbits; G4-V, pomegranate peel extract treatment of V-line rabbits; G5-V, combined treatment of V-line rabbits. Data obtained from 5 rabbits/group. Means carrying ^a–d^ significantly differ among different groups of the same row, while means carrying ^A,B^ significantly differ among New Zealand and V-line of the same row.

**Table 8:** Economic efficiency of the New Zealand white and V-line rabbits in response to coccidial infection and herbal extract treatment.

Variables	New Zealand White Rabbits						V-Line Rabbits						SEM	*p*-Value		
	G1-N	G2-N	G3-N	G4-N	G5-N	Overall	G1-V	G2-V	G3-V	G4-V	G5-V	Overall		B	G	B × G
Rabbit price (LE)	30	30	30	30	30	30	35	35	35	35	35		-	-	-	-
Labor (LE)	2	2	2	2	2	2	2	2	2	2	2	2	-	-	-	-
Water and electricity (LE)	0.5	0.5	0.5	0.5	0.5	0.5	0.5	0.5	0.5	0.5	0.5	0.5	-	-	-	-
Herbal extract (LE)	0	0	0	5	5	2	0	0	0	5	5	2	-	-	-	-
TVM (LE)	6.4	6.4	6.4	11.4	11.4	8.4	6.4	6.4	6.4	11.4	11.4	8.4	-	-	-	-
Feed cost (LE)	26.69 ^b^	24.15 ^b,c^	24.59 ^b,c^	23.59 ^b,c^	22.95 ^c^	24.39 ^A^	30.57 ^a^	23.92 ^b,c^	24.95 ^b,c^	23.99 ^b,c^	24.02 ^b,c^	25.49 ^A^	0.39	NS	**	NS
TVC (LE)	65.59 ^b,c,d^	63.05 ^d^	63.49 ^c,d^	67.49 ^b^	66.85 ^b,c^	65.29 ^B^	74.47 ^a^	67.82 ^b^	68.85 ^b^	72.89 ^a^	72.92 ^a^	71.39 ^A^	0.39	**	**	NS
TFC (LE)	3.25	3.25	3.25	3.25	3.25	3.25	3.25	3.25	3.25	3.25	3.25	3.25				
TC (LE)	68.84 ^b,c,d^	66.30 ^d^	66.74 ^c,d^	70.74 ^b^	70.10 ^b,c^	68.54 ^B^	77.72 ^a^	71.07 ^b^	72.10 ^b^	76.14 ^a^	76.17 ^a^	74.64 ^A^	0.39	**	**	NS
TR (LE)	87.72 ^c,d^	72.16 ^f^	82.79 ^d,e^	82.09 ^d,e^	81.78 ^d,e^	81.31 ^B^	112.89 ^a^	80.69 ^e^	94.71 ^b^	92.44 ^b,c^	93.86 ^b,c^	94.92 ^A^	0.73	**	**	**
NP (LE)	18.88 ^b,c^	5.86 ^f^	16.05 ^c,d,e^	11.35 ^e,f^	11.68 ^d,e,f^	12.76 ^B^	35.17 ^a^	9.62 ^f^	22.61 ^b^	16.30 ^c,d,e^	17.69 ^b,c,d^	20.28 ^A^	0.70	**	**	NS
Capital turnover	1.27 ^b,c^	1.09 ^f^	1.24 ^b,c,d^	1.16 ^d,e,f^	1.17 ^d,e,f^	1.19 ^B^	1.45 ^a^	1.14 ^e,f^	1.31 ^b^	1.21 ^c,d,e^	1.23 ^b,c,d^	1.27 ^A^	0.01	**	**	NS
Return on investment	0.27 ^b,c^	0.09 ^f^	0.24 ^b,c,d^	0.16 ^d,e,f^	0.17 ^d,e,f^	0.19 ^B^	0.45 ^a^	0.14 ^e,f^	0.31 ^b^	0.21 ^c,d,e^	0.23 ^b,c,d^	0.27 ^A^	0.01	**	**	NS
TR% to control negative	100.01 ^a^	82.27 ^c^	94.39 ^a,b^	93.59 ^b^	93.24 ^b^	92.70 ^A^	100.00 ^a^	71.48 ^d^	83.89 ^c^	81.89 ^c^	83.15 ^c^	84.08 ^B^	0.69	**	**	NS
TR% to control positive	121.56 ^b^	100.00 ^d^	114.74 ^b,c^	113.76 ^b,c^	113.34 ^c^	112.68 ^B^	139.91 ^a^	100.00 ^d^	117.37 ^b,c^	114.56 ^b,c^	116.32 ^b,c^	117.63 ^A^	0.93	*	**	*
NP% to control negative	100.00 ^a^	31.04 ^d,e^	85.02 ^a,b^	60.11 ^c^	61.85 ^c^	67.61 ^A^	100.00 ^a^	27.36 ^e^	64.28 ^b,c^	46.35 ^c,d,e^	50.31 ^c,d^	57.66 ^A^	2.47	NS	**	NS
NP% to control positive	322.17 ^a,b^	100.02 ^e^	273.89 ^b,c^	193.66 ^d^	199.27 ^d^	217.80 ^A^	365.56 ^a^	100.04 ^e^	235.01 ^c,d^	169.45 ^d,e^	183.93 ^d^	210.80 ^A^	8.54	NS	**	NS

TVM, total veterinary management; TVC, total variable cost; TFC, total fixed cost; TC, total cost; TR, total return; NP, net profit. B, breed effect; G, group effect; B × G, breed and group interaction; * *p* ≤ 0.05, ** *p* ≤ 0.01, NS (non-significant): *p* > 0.05. G1-N, negative control group of New Zealand rabbits; G2-N, positive control group of New Zealand rabbits; G3-N, neem leaf extract treatment of New Zealand rabbits; G4-N, pomegranate peel extract treatment of New Zealand rabbits; G5-N, combined treatment of New Zealand rabbits; G1-V, negative control group of V-line rabbits; G2-V, positive control group of V-line rabbits; G3-V, neem leaf extract treatment of V-line rabbits; G4-V, pomegranate peel extract treatment of V-line rabbits; G5-V, combined treatment of V-line rabbits. Means carrying ^a–f^ significantly differ among different groups of the same row, while means carrying ^A,B^ significantly differ among New Zealand and V- line of the same row.

## Supplementary Materials

The following are available online at https://www.mdpi.com/article/10.3390/ani11082441/s1, Figure S1: Light micrographs of the sporulated oocyst of three species of Eimeria collected from naturally infected rabbit “A; E. intestinalis (67%), B; E. magna (22%) and C; E. media (11%)”. Figure S2: Clinical findings of the infected rabbits with Eimeria spp. showing (A) depression, distended abdomen, and (*) presence of very moist feces (undefined shape and texture); (B) dull, rough coat with dirty soiled perineum and extremities with watery feces (b) Perineum of the herbal treated rabbit appeared clean and expel well-formed feces. Table S1: Experimental design and different treatments.
