# Supplementary material for: Ameliorative Effect of Neem Leaf and Pomegranate Peel Extracts in Coccidial Infections in New Zealand and V-Line Rabbits: Performance, Intestinal Health, Oocyst Shedding, Carcass Traits, and Effect on Economic Measures
Source: Animals (Basel). 2021 Aug 19;11(8):2441. doi: 10.3390/ani11082441 (PMC8388781; doi:10.3390/ani11082441)
Supplement: Supplementary file 1 [file animals-11-02441-s001.zip › animals-1311557-supplementary.pdf]

Table S1. Experimental design and different treatments.

Breed	Experiment Groups		Coccidial infection	Herbal Treatments [*]	
	Name	Code		Neem extract	Pomegranate extract
New Zealand white Breed	Control negative	G1-N	-	-	-
	Control positive	G2-N	+	-	-
	Aqeous Neem extract treatment [ǂ]	G3-N	+	+	-
	Ethanolic Pomegranate extract treatment [*]	G4-N	+	-	+
	Combined treatment [ǂ*]	G5-N	+	+	+
V- line Breed	Control negative	G1-V	-	-	-
	Control positive	G2-V	+	-	-
	Aqeous Neem extract treatment [ǂ]	G3-V	+	+	-
	Ethanolic Pomegranate extract treatment [*]	G4-V	+	-	+
	Combined treatment [ǂ*]	G5-V	+	+	+

Dose of *Eimeria* spp. infection 5 X 10^4/ rabbit .Dose of neem extract 50 ml/ litre drinking water, Dose of pomegranate extract 300mg/ kg BW for 6 days

Figure S1. Light micrographs of the sporulated oocyst of three species of Eimeria collected from naturally infected rabbit "A; E. intestinalis (67%), B; E. magna (22%) and C; E. media (11%)".

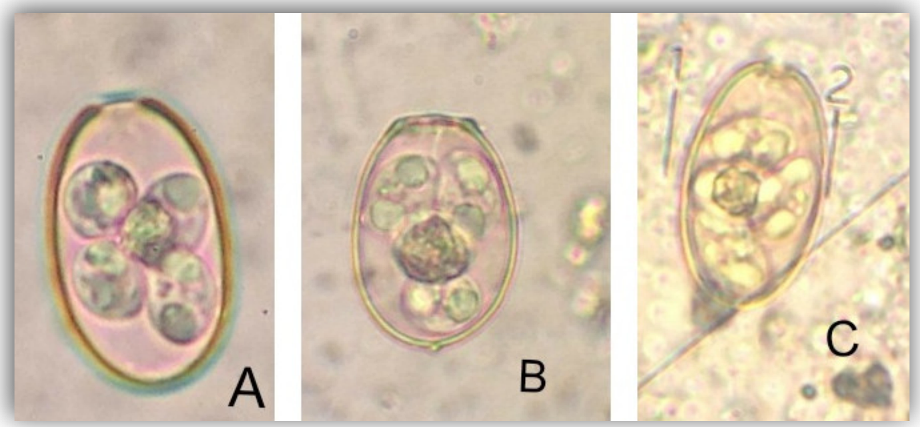

Figure S2: Clinical findings of the infected rabbits with Eimeria spp. showing (A) depression, distended abdomen, and (*) presence of very moist feces (undefined shape and texture); (B) dull, rough coat with dirty soiled perineum and extremities with watery feces (b) Perineum of the herbal treated rabbit appeared clean and expel well-formed feces.

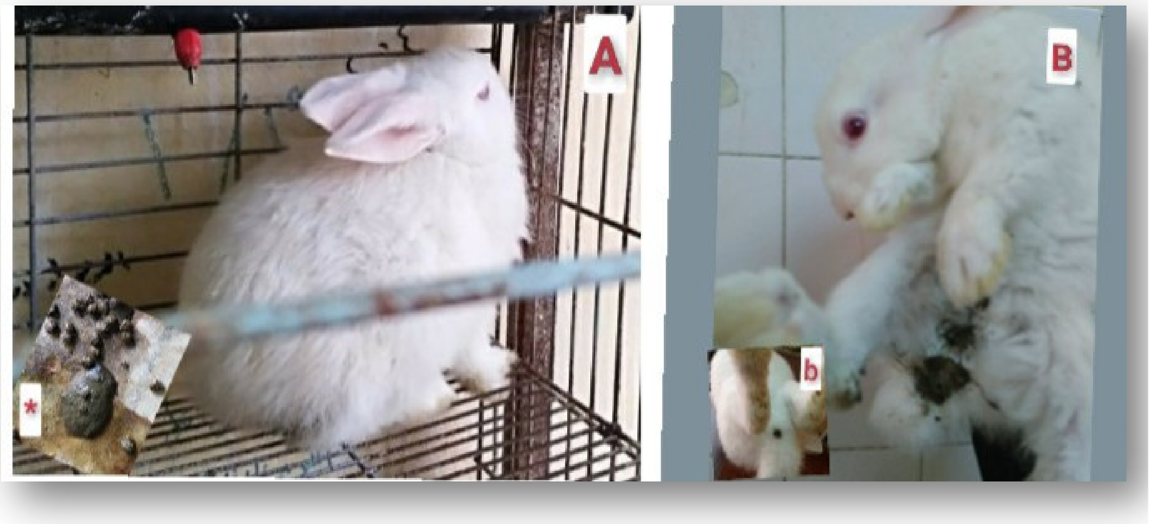
A
*
B
b
